# An Electrochemical Sensor Based on Gold and Bismuth Bimetallic Nanoparticles Decorated L-Cysteine Functionalized Graphene Oxide Nanocomposites for Sensitive Detection of Iron Ions in Water Samples

**DOI:** 10.3390/nano11092386

**Published:** 2021-09-14

**Authors:** Na Zhou, Jing Li, Shaoxia Wang, Xuming Zhuang, Shouqing Ni, Feng Luan, Xuran Wu, Shunyang Yu

**Affiliations:** 1 Shandong Key Laboratory of Coastal Environmental Processes, Yantai Institute of Coastal Zone Research, Chinese Academy of Sciences, Yantai 264003, China; nzhou@yic.ac.cn; 2 College of Chemistry and Chemical Engineering, Yantai University, Yantai 264005, China; lj508837@163.com (J.L.); 18865557620@163.com (S.W.); fluan@sina.com (F.L.); ytdxwxr@126.com (X.W.); 3 Shandong Provincial Key Laboratory of Water Pollution Control and Resource Reuse, School of Environmental Science and Engineering, Shandong University, Qingdao 266237, China; sqni@sdu.edu.cn

**Keywords:** bimetallic, iron ions, gold, bismuth, graphene oxide, electrochemical sensor

## Abstract

In this work, gold and bismuth bimetallic nanoparticles decorated L-cysteine functionalized graphene oxide nanocomposites (Au-BiNPs/SH-GO) were prepared and applied to selective detection of Fe(III) in lake and seawater samples by modifying onto glassy carbon electrodes. Bimetallic nanoparticles have various excellent properties and better catalytic properties because of the unique synergistic effect between metals. The modified electrode was characterized by scanning electron microscopy, energy dispersive X-ray spectroscopy, X-ray diffraction, X-ray photoelectron spectroscopy, Fourier transform infrared spectroscopy, and Raman spectroscopy. Under optimized conditions, current peak intensity increased linearly with increasing Fe(III) concentration over the range of 0.2–50 μM and a detection limit of 0.07 μM (S/N = 3). The Au-BiNPs/SH-GO/GCE was used for the determination of Fe(III) in lake and seawater samples with recoveries ranged from 90 to 103%. Those satisfactory results revealed the potential application of the Au-BiNPs/SH-GO electrochemical sensor for heavy metals detection in environmental monitoring.

## 1. Introduction

Iron is the fourth highest element in the crust with a total content of about 5% [1]. Iron is an indispensable micronutrient element in the growth of plants and animals [2,3,4]. Iron itself is not toxic, but it can cause iron poisoning with an overdose of iron preparations. Excessive Fe(III) solution in drinking water could lead to a lot of problems to the people’s health. The suggested guideline level of iron ions in water is 0.3 ppm [5]. Hence, it is very important to study the iron ion detection problem in environmental water. Hence, iron ion levels are necessarily determined in different fields, such as clinical, drug, and environmental monitoring [6,7,8,9,10,11,12]. In the past few decades, a large number of methods have been developed and applied to detect iron ions, including flame atomic absorption spectrometry [13], inductively coupled plasma mass spectrometry [14], reversed-phase high-performance liquid chromatography [15], electrochemistry [16,17], colorimetric analysis [18,19,20], and fluorescent spectrophotometry [21,22,23]. Among these analytical methods, electrochemical analysis has attracted much attention owing to its simple and fast operation, high sensitivity and low cost [24].

In order to improve the selectivity and sensitivity of the electrode, the chemical modification of the electrode surface becomes the focus of interest in research [25]. The researchers used nanomaterials modified electrodes to construct electrochemical sensors, considering their unique surface effects, small size and excellent photoelectric properties. The large comparative area and strong adsorption properties of nanomaterials can achieve adsorption equilibrium with a short time in the detection of metal ions, which is conducive to the enrichment and detection of metal ions in solution.

In recent years, bimetallic nanoparticles have been widely studied because of their special catalysis, electrical and optical properties [26,27]. Unlike single nanomaterials, bimetallic nanoparticles have many more excellent properties than single component materials, which fills up the shortcomings of single component materials. In addition, bimetallic nanoparticles can produce comprehensive effects on the basis of maintaining their own excellent properties [28]. Gold and bismuth bimetallic nanoparticles (Au-BiNPs) have small nanometer size, strong catalytic performance and excellent dielectric properties. The properties of Au-BiNPs are better than those of gold or bismuth single nanoparticles, and they can form amalgam alloys like heavy metal ions [29]. Moreover, it has the advantages of low background current and good stability, which is beneficial to the high sensitivity analysis of heavy metal ions.

Graphene oxide (GO) has been widely used due to its unique structure, good electrical conductivity, strong mechanical properties, and large surface area [30,31]. Ding et al. functionalized graphene by introducing different metal nanoparticles or organic molecules on the surface of graphene to improve the detected effects of Hg(II) and As(II) [32,33]. The functional groups on mercapto-modified graphene oxide (SH-GO) adsorb heavy metals by forming chelates with metal ions, which makes the nanoparticles loaded better and improves the electron transfer efficiency between materials and electrodes [34]. Sitko et al. [35] used quantitative adsorption of Co(II), Ni(II), Cu(II), As(III), Cd(II), and Pb(II) by the wrinkled structure of SH-GO nanosheets.

In this paper, gold and bismuth bimetallic nanoparticles decorated with L-cysteine functionalized graphene oxide nanocomposites (Au-BiNPs/SH-GO) were prepared and applied to selective detection of Fe(III) in lake and seawater samples by modifying them on a glassy carbon electrode using the square wave voltammetry method (SWV). The sensor is very prominent with low detection, high sensitivity, and wide linear range. The Au-BiNPs/SH-GO/GCE was also successfully applied for the detection of total dissolved iron ions in water samples with satisfactory results.

## 2. Experimental Details

### 2.1. Materials

L-cysteine, 3-ammonia propyl-3-ethoxy silane, *N*,*N*′-dicyclohexylcarbodiimide (DCC), *N*,*N*-Dimethylformamide (DMF), potassium ferricyanide (K_3_[Fe(CN)_6_]), potassium chloride (KCl), and ethanol were purchased from Sinopharm Chemical Reagent Co., Ltd. (Beijing, China). Chloroauric acid (HAuCl_4_), Bismuth nitrate pentahydrate (BiNO_3_·5H_2_O) and Sodium borohydride (NaBH_4_) were purchased from Medicine Group Chemical Reagent Co., Ltd. (Shanghai, China). GO was obtained from the XFNano Materials Technology Company (Nanjing, China). All chemicals were analytical grade and used as received. Ultrapure water (18.2 MΩ cm) was used in all experiments.

### 2.2. Apparatus

Scanning electron microscopy (SEM) images were obtained using a field emission scanning electron microanalyzer (S-4800, Hitachi, Tokyo, Japan). Energy dispersive X-ray spectroscopy (EDX) was measured by EDX HORIBA EX-350. X-ray diffraction (XRD) measurements were obtained using an X-ray diffractometer to determine the crystal phase composition of the sample (XRD6000, Shimadzu, Tokyo, Japan). X-ray photoelectron spectra (XPS) analysis was tested with an ESCALAB 250Xi (Thermo, Waltham, MA, USA). All electrochemical experiments were carried out on a workstation (CHI660C, Shanghai Chenhua Instrument Co., Ltd., Shanghai, China). Bare or modified electrodes were used as working electrodes, and a Pt wire electrode was the auxiliary electrode. An Ag/AgCl (saturated KCl solution) was used as a reference electrode.

### 2.3. Preparation of Au-BiNPs/SH-GO Nanomaterials

The suspension of GO (0.2 g) in anhydrous ethanol (30 mL) was sonicated for 1 h. Then, 3-ammonia propyl-3-ethoxy silane (2 mL) was added and refluxed at 70 °C for 8 h. The obtained product was purified by anhydrous ethanol and dried for 10 h. Moreover, the suspension of resulted powder (0.2 g) in DMF (50 mL) was sonicated for 15 min and DCC (0.1 g) with L-cysteine (0.1 g) was added to the suspension and refluxed at 70 °C for 24 h. The obtained product was purified by anhydrous ethanol and dried for 10 h again. Finally, the material SH-GO was obtained [36].

The SH-GO was uniformly dispersed into DMF, then the aqueous solution of BiNO_3_·5H_2_O and HAuCl_4_ was added and mixed. The final concentration of Bi(III) and Au(III) was 0.01 M. The content of GO was 1.5% of the content of BiNO_3_·5H_2_O (weight percentage), and then the pH of the solution was adjusted by NaOH to 12. The mixed solution was transferred to the reactor of PTFE by stirring for 30 min, and the reaction was heated at 120 °C for 12 h. Finally, the nanocomposites were placed on the cold trap of the vacuum freeze dryer to pre freezing for 3 h at −30 °C. Then, the frozen nanocomposites were put in a drying chamber with the chamber was evacuated less than 100 Pa for 8 h.

### 2.4. Electrode Preparation and Modification

**Scheme 1:**
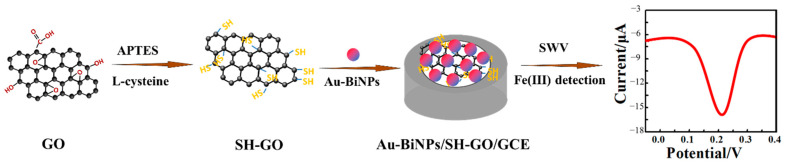
Schematic diagram for the synthesis of Au-BiNPs/SH-GO/GCE and its application in detection of Fe(III).

First, the GCE was polished with 0.5 and 0.05 µm diameter alumina polishing powder. Then, the polished electrodes were then washed in ethanol and distilled water for 3 min and dried in air. Au-BiNPs/SH-GO (2.0 mg) was dispersed in 1.0 mL of distilled water. The dispersed liquid droplets of 5.0 µL nanomaterials were coated on the surface of GCE, and then dried under an infrared lamp for half an hour, rinsed with ultrapure water several times to obtain Au-BiNPs/SH-GO/GCE (Scheme 1). IR lamp was used for the nanocomposite drying fast and heating evenly on the electrode surface. The ultrapure water treatment was in order to remove excess unabsorbed nanomaterials. The mercapto-modified graphene oxide material modified GCE (SH-GO/GCE) and graphene oxide material modified GCE (GO/GCE) used the same method of preparation.

### 2.5. Electrochemical Detection Process

The electrochemical detection process was carried out in 0.1 M HCl supporting electrolyte. SWV was used to detect the concentration of Fe(III) in the modified electrodes by measuring the reduction current of Fe(III) reduced to Fe(II) on the surface of the modified electrodes. In the detection process, the three-electrode system was inserted into the Fe(III) electrolyte containing a certain concentration. After enrichment, the negative sweep electrode potential of Fe(III) on Au-BiNPs/SH-GO/GCE was obtained under the potential window of −0.2 to 0.6 V. The experimental parameters are as follows: the initial potential is 0.6 V, the termination potential is −0.2 V, the amplitude is 0.025 V, the frequency is 15 Hz, and the equilibrium time is 2 s.

## 3. Results and Discussion

### 3.1. Structural and Compositional Characterizations of Au-BiNPs/SH-GO/GCE

**Figure 1:**
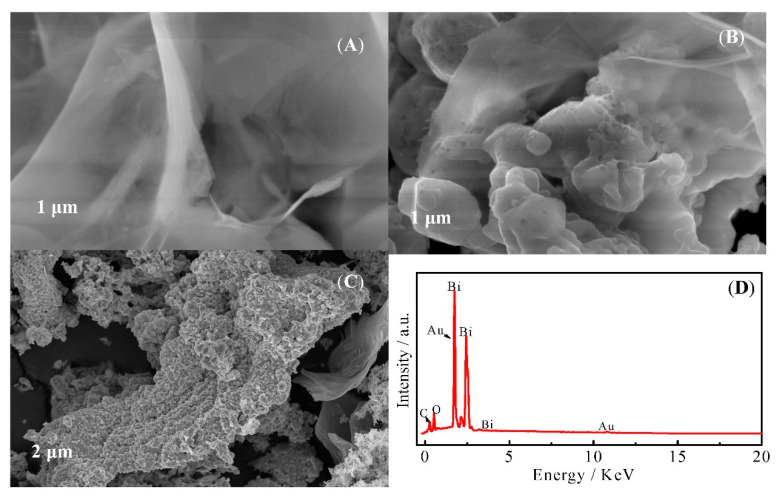
SEM images of (**A**) GO/GCE; (**B**) SH-GO/GCE and (**C**) Au-BiNPs/SH-GO/GCE; (**D**) EDX patterns of Au-BiNPs/SH-GO/GCE.

The surface morphologies of the various modified electrodes were characterized by SEM and EDX (Figure 1). Compared with the surface of the GO/GCE (Figure 1A), SH-GO/GCE (Figure 1B) surface showed more folds [37], and these large specific surface areas provide a larger adhesion area for the loading of metal nanoparticles. At the same time, it is also beneficial to the complete contact between electrolyte and electrode surface [38]. It is showed that SH-GO/GCE has been successfully combined with gold and bismuth nanoparticles (Figure 1C). SH-GO had lots of sulfhydryl groups and wrinkles to lead to strong chelation with Fe(III) [34], and the Au-BiNPs could combine with metal ions to form amalgam analogs [29]. The nanomaterial of SH-GO was combined with gold and bismuth nanoparticles not only provides a large specific surface area and binding sites but also improves the electrochemical signal for Fe(III) detection. Combined with the EDX analysis of Au-BiNPs/SH-GO/GCE in Figure 1D, the results showed that the main components of Au-BiNPs/SH-GO/GCE are four elements of C, O, Au, Bi. The C element mainly comes from the skeleton structure of SH-GO/GCE, while the O element mainly comes from the oxygen-containing functional groups such as carboxyl, hydroxyl, epoxide, and carbonyl groups on the surface of SH-GO/GCE. Sulfur has a response signal in 2.4 KeV [39], which may be covered by other signal peaks, so it needs to be analyzed by other characterization methods. However, a more detailed analysis of the chemical composition of the surface of Au-BiNPs/SH-GO/GCE and the presence of C, S, Au, and Bi by elemental mapping taken with EDX is shown in Figure S1. Therefore, it is proved that the elements are uniformly distributed in the nanomaterials.

**Figure 2:**
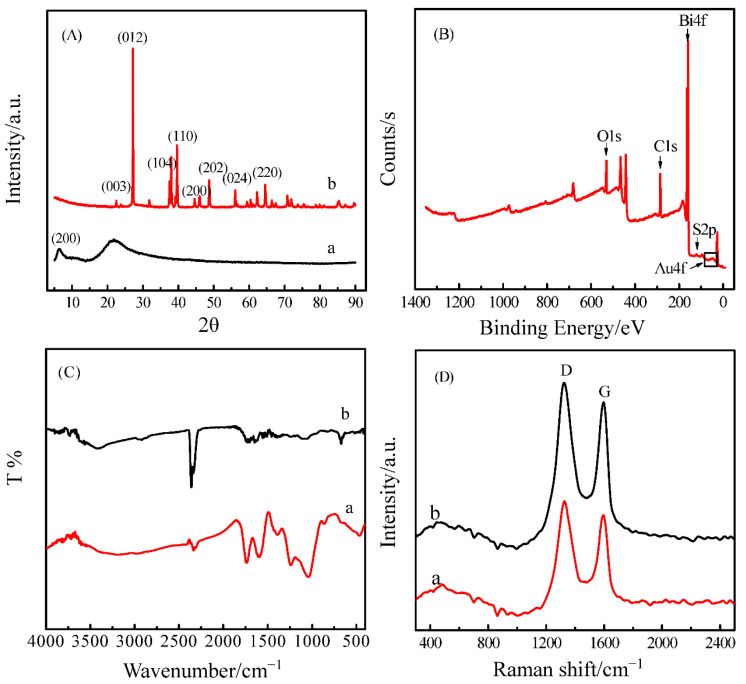
(**A**) XRD patterns of (a) SH-GO/GCE, (b) Au-BiNPs/SH-GO/GCE; (**B**) XPS survey spectrum of Au-BiNPs/SH-GO/GCE; (**C**) FT-IR spectra of (a) SH-GO/GCE, (b) Au-BiNPs/SH-GO/GCE; and (**D**) Raman spectra of (a) SH-GO/GCE, (b) Au-BiNPs/SH-GO/GCE.

Figure 2A shows the XRD spectra of SH-GO/GCE (a) and Au-BiNPs/SH-GO/GCE (b). Compared with the peak at 10.7° of GO/GCE, the order of wide peak arrangement of SH-GO/GCE at 24.6° was worse. The results showed that the elimination of functional groups leads to the decrease of layer spacing and the (200) plane is S crystal structure plane. The activity peak of Au-BiNPs/SH-GO/GCE at 10.7° graphene is not obvious. These peaks at 27.2°, 38.1°, and 39.7° belong to the (012), (104), and (110) planes of the rhombohedral Bi crystal structure. In addition, the characteristic peaks at 44.4° and 64.5° were corresponding to (200) and (220) Au structure planes [40].

XPS spectrum of Au-BiNPs/SH-GO/GCE (Figure 2B and Figure S2) showed the existence of five elements, C, Au, Bi, S, and O, respectively. Figure S2A showed the presence of three main types of carbon bonds of C–C (284.7 eV), C–O (285.6 eV) and C=O (288.9 eV). As shown in Figure S2B, the peak at 84.0 eV was caused by Au4f_7/2_, while the Au4f_5/2_ signal peak appears at 87.76 eV. Meanwhile, the peak at 153.7 eV and 157.7 eV were caused by Bi4f (Figure S2C) and S2p (Figure S2D), respectively. The results confirm the presence of Au, Bi nanoparticles and sulfhydryl group in the Au-BiNPs/SH-GO/GCE [41]. The XPS spectrum clearly evidenced the synthesis of Au-BiNPs/SH-GO nanocomposites.

The Fourier transform infrared spectra (FT-IR) are shown in Figure 2C. The peaks at 1734, 1616, 1390 and 1050 cm^−1^ may be caused by the stretching vibration of –COOH, O–H, C–OH, and C–O–C functional groups [42]. It is indicated that there are many oxygen-containing functional groups on the surface of SH-GO/GCE. In addition, the –COOH, C–OH, and C–O–C groups were also found in Au-BiNPs/SH-GO/GCE, but the peak of C–O–C was obviously weakened. It is clearly demonstrated that the formation of metal nanoparticles on graphene oxide lamellae can promote the reduction of graphene oxide.

At the same time, Raman spectroscopy was used to characterize the structure of Au-BiNPs/SH-GO/GCE (Figure 2D). Both SH-GO/GCE and Au-BiNPs/SH-GO/GCE have two distinct characteristic peaks. The G peak near 1581 cm^−1^ is the characteristic peak of carbon sp^2^, which reflects its symmetry and degree of crystallization. The D peak near 1332 cm^−1^ is the defect peak, which reflects the disorder and defect degree of the graphite sheet. The I_D_/I_G_ value of Au-BiNPs/SH-GO/GCE (1.34) was higher than 0.97 of SH-GO/GCE, suggesting the generation of new quasi-amorphous sp^2^-bonded carbons. Combined with the above characterization methods, we successfully obtained Au-BiNPs/SH-GO nanocomposites.

### 3.2. Electrochemical Characterization of the AuNPs/SH-GO/GCE

**Figure 3:**
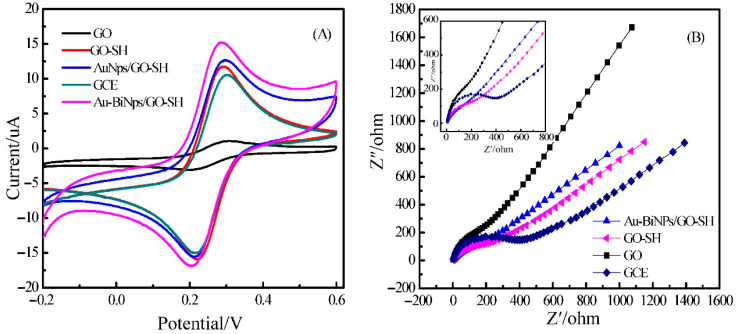
(**A**) CVs of different electrodes in 1 mM [Fe(CN)_6_]^3−/4−^ solution containing 0.1 M KCl for GCE, GO/GCE, SH-GO/GCE, AuNPs/SH-GO/GCE and Au-BiNPs/SH-GO/GCE; (**B**) Nyquist plots of different electrodes in 1 mM [Fe(CN)_6_]^3−/4−^ solution containing 0.1 M KCl for GCE, GO/GCE, SH-GO/GCE, AuNPs/SH-GO/GCE and Au-BiNPs/SH-GO/GCE.

Several electrochemical techniques of cyclic voltammetry (CV) and electrochemical impedance spectroscopy (EIS) were applied to investigate the features of Au-BiNPs/SH-GO/GCE. CV was implemented in 5 mM [Fe(CN)_6_]^3−/4−^ containing 0.1 M KCl, as shown in Figure 3A. Compared with the GCE and SH-GO/GCE, the redox peak currents of Au-BiNPs/SH-GO/GCE were, respectively increased about 35% and 25%, indicating this sensor has better electrochemical catalytic sites and performance [43]. The results showed that Au-BiNPs/SH-GO/GCE has a better electronic response signal, which is beneficial to the determination of metal ions.

The EIS curves are presented in Figure 3B. It is showed that the semicircle of the GCE electrode is slightly larger than that of the Au-BiNPs/SH-GO/GCE electrode, indicating that the charge transfer resistance on the Au-BiNPs/SH-GO/GCE electrode is smaller due to the modified metal nanoparticles increase the speed of electron transfer. The above results further prove that the charge transfer rate of Au-BiNPs/SH-GO/GCE electrode is higher than that of GCE, GO, and Au-BiNPs/SH-GO/GCE electrode.

### 3.3. Optimization of Supporting Electrolytes

Fe(III) is easy to hydrolyze under neutral or alkaline conditions, so the supporting electrolyte used to detect Fe(III) should be an acidic solution. In this experiment, HAc-NaAc, H_2_SO_4_, HNO_3_ and HCl with a concentration of 0.1 M were selected as supporting electrolytes. The reduction current of Fe(III) in different electrolytes was investigated under the same experimental conditions. The experimental results show that Fe(III) has the largest current response in HCl, followed by H_2_SO_4_, while the reduction peak current in HAc-NaAc and H_2_SO_4_ is very small, as shown in Figure S3A. Finally, HCl was chosen as the supporting electrolyte because the Cl^−^ in HCl could act as a salt bridge in the solution and accelerate the electron transfer between Fe(III) and electrode. In addition, different concentrations of HCl were optimized, as shown in Figure S3B. When the concentration of HCl was 0.1 M, the maximum current response signal is obtained. Therefore, 0.1 M HCl was chosen as the supporting electrolyte in this experiment.

### 3.4. Electrochemical Sensing of Fe(III) on Au-BiNPs/SH-GO/GCE

**Figure 4:**
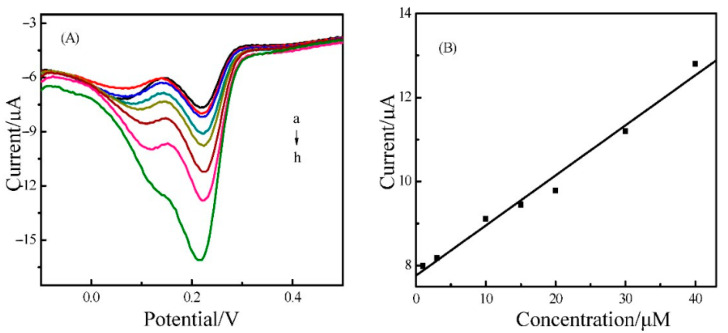
(**A**) SWV curves of Au-BiNPs/SH-GO/GCE in 0.1 M HCl containing different Fe(III) concentrations of 0.2, 0.8, 1, 5, 10, 20, 30, and 50 μM for a to h. (**B**) Linear plot of peak current versus Fe(III) concentration.

Under the optimum conditions, the standard iron ions solution was detected by SWV with Au-BiNPs/SH-GO/GCE modified electrode. Figure 4A shows typical SWV curves with a fast response performance. The electrode has a good linearity for the detection of Fe(III) in the range of 0.2 μM to 50 μM. The linear regression equation (Figure 4B) is I (µA) = 0.1191*c* (µM) + 7.764 (R^2^ = 0.993) with the detection limit of 0.07 μM (S/N = 3). The results showed that the Au-BiNPs/SH-GO/GCE has a better sensitivity to the Fe(III). Compared with other previously reported sensors for Fe(III) detection (Table 1) [44,45,46,47,48,49], the proposed sensing platform processed more sensitivity and lower limit of detection.

### 3.5. Stability, Repeatability and Selectivity of the Sensor

The stability, repeatability and selectivity of Au-BiNPs/SH-GO/GCE were also investigated. The stability of the electrode was measured 10 times by the same Au-BiNPs/SH-GO/GCE electrode in Fe(III) (1 μM) solution. The relative standard deviation (RSD) of the peak current was 1.67%. The repeatability of the electrode was measured by 10 modified electrodes in Fe(III) (1 μM) solution revealed the RSD of 2.35%. It is indicated that Fe(III) detection by Au-BiNPs/SH-GO/GCE has good stability and reproducibility.

**Figure 5:**
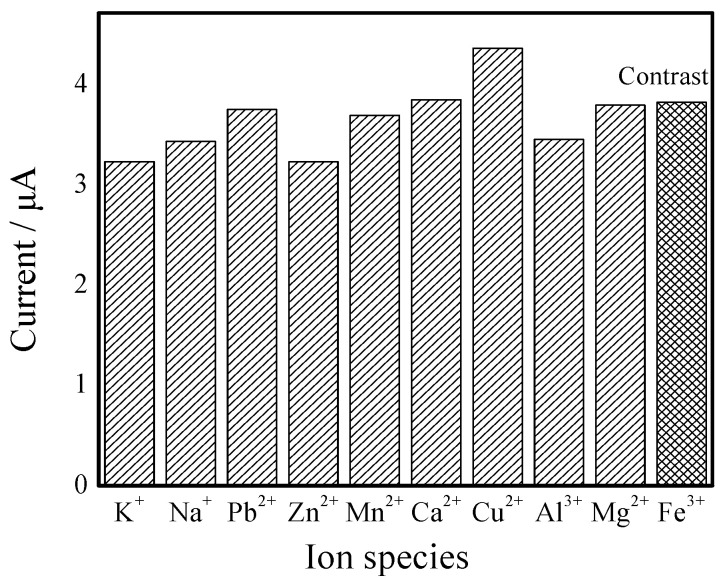
Effects of various interference metal ions on the electrochemical signals of Fe(III) at Au-BiNPs/SH-GO/GCE in 0.1 M HCl containing 1 μM Fe(III) in the presence of 50-fold concentrations of Zn^2+^, Mn^2+^, Ca^2+^, Al^3+^ and 100-fold concentrations of K^+^, Na^+^, Pb^2+^, Mg^2+^, Cu^2+^.

Furthermore, the influence of common co-existing metal ions, such as K^+^, Na^+^, Pb^2+^, Zn^2+^, Mn^2+^, Ca^2+^, Cu^2+^, Al^3+^, Mg^2+^ on the detection of Fe(III) was tested under the optimized conditions. The results showed (Figure 5) that 50-fold concentrations of Zn^2+^, Mn^2+^, Ca^2+^, Al^3+^ and 100-fold concentrations of K^+^, Na^+^, Pb^2+^, Mg^2+^, Cu^2+^ had no significant effect on the determination of Fe(III) when the concentration of Fe(III) was 1 μM.

## 4. Detection Application in Lake Water and Seawater Samples

The applicability of the sensor in lake and seawater samples was studied. All water samples were filtered with 0.45 μm membrane filter and then digested by microwave. The water samples were diluted 10 times, then added to 0.1 M HCl for detection. The concentration of Fe(III) in water samples was obtained by the standard addition method. As shown in Table 2, it is found that the Fe(III) concentration measured by the sensor is in good agreement with the results of ICP-MS and the recoveries ranged between 93 and 103% with the RSD lower than 2.7%. The results show that the sensor can be used for the daily monitoring of Fe(III) and has significant application prospects.

## 5. Conclusions

An Au-BiNPs/SH-GO/GCE electrochemical sensor has been developed which could be used for the detection of Fe(III). These nanomaterials were characterized by SEM, EDX, XRD, XPS, FT-IR, Raman, CV, and EIS, demonstrating SH-GO could carry many mercapto groups and possess better stability and dispersibility than GO. Moreover, gold and bismuth bimetallic nanoparticles had excellent ability to absorb and detect Fe(III). Experimental results indicated that the Au-BiNPs/SH-GO/GCE sensor has beneficial properties of high sensitivity, excellent stability, and reproducibility. Au-BiNPs/SH-GO/GCE sensor is also used for the detection of Fe(III) in lake water and seawater samples with correct and reliable results. The nanocomposites can also be used in other areas with great potential for research in the future. 

## Figures and Tables

**Table 1:** Comparison of this work with previously reported Fe(III) detection methods.

Modified Nanomaterials	Linear Range (μM)	Detection Limit (μM)	Ref.
FAQT ^a^	5–100	0.23	[1]
P(TPP) ^b^	0.1–100	0.173	[2]
DPYBT ^c^	3.8–7.2	3.04	[3]
GQD/PS-AER ^d^	1–7	0.65	[4]
CPB@PSAA ^e^	5–150	2.2	[5]
AgNCs-PBH ^f^	0.14–27	0.045	[6]
Au-BiNPs/SH-GO	0.5–50	0.07	This work

^a^ ferrocene appended quinoline-triazole; ^b^ pyrene-substituted poly(2,5-dithienylpyrrole); ^c^ 4,7-di-pyrrol-1-yl-benzo[1,2,5]thiadiazole; ^d^ graphene quantum dots/polystyrenic anion-exchange resin; ^e^ CsPbBr_3_ perovskite quantum dots@poly(styrene/acrylamide); ^f^ silver nanoclusters@poly vinyl alcohol-borax hydrogel.

**Table 2:** Assay results of Fe(III) in samples of lake water and seawater.

Samples	Added (µM)	Detected (µM)	ICP-MS (µM)	Recovery (%, *n* = 5)	RSD (%)
Lake water	0	4.04 ± 0.08	4.10	-	1.98
	0.3	4.32 ± 0.12	4.42	93.3	2.77
	3	6.86 ± 0.07	7.01	94.0	1.02
	30	35.07 ± 0.20	35.33	103.4	0.57
Seawater	0	2.15 ± 0.03	2.08	-	1.40
	0.3	2.42 ± 0.04	2.37	90.0	1.65
	3	5.16 ± 0.11	5.14	100.3	2.13
	30	31.87 ± 0.64	31.85	98.2	2.01

## Supplementary Materials

The following are available online at https://www.mdpi.com/article/10.3390/nano11092386/s1, Figure S1: Elemental mapping of C, Au, S, and Bi element distribution, respectively, Figure S2: XPS survey spectrum of (A) C1s (B) Au4f (C) Bi4f and (D) S2p, Figure S3: (A) SWV measurements of 5 μM Fe(III) at Au-BiNPs/SH-GO/GCE in different electrolytes. 0.1 M HAc-NaAc (a), 0.1 M HNO_3_ (b), 0.1 M H_2_SO_4_ (c) and 0.1 M HCl (d); (B) Effect of the concentration of HCl solution on the peak of Fe(III) at Au-BiNPs/SH-GO/GCE.
