# Supplementary material for: An Electrochemical Sensor Based on Gold and Bismuth Bimetallic Nanoparticles Decorated L-Cysteine Functionalized Graphene Oxide Nanocomposites for Sensitive Detection of Iron Ions in Water Samples
Source: Nanomaterials (Basel). 2021 Sep 14;11(9):2386. doi: 10.3390/nano11092386 (PMC8466943; doi:10.3390/nano11092386)
Supplement: Supplementary file 1 [file nanomaterials-11-02386-s001.zip › nanomaterials-1369799-supplementary.pdf]

-*Supporting information*-

An Electrochemical Sensor Based on Gold and Bismuth Bimetallic Nanoparticles Decorated L-Cysteine Functionalized Graphene Oxide Nanocomposites for Sensitive Detection of Iron Ions in Water Samples

Na Zhou [1], Jing Li [2], Shaoxia Wang [2], Xuming Zhuang [2,*], Shouqing Ni [3], Feng Luan [2], Xuran Wu [2] and Shunyang Yu [1,*]

[1] Shandong Key Laboratory of Coastal Environmental Processes, Yantai Institute of Coastal Zone Research, Chinese Academy of Sciences, Yantai 264003, China; nzhou@yic.ac.cn

[2] College of Chemistry and Chemical Engineering, Yantai University, Yantai 264005, China; lj508837@163.com (J.L.); 18865557620@163.com (S.W.); fluan@sina.com (F.L.); ytdxwxr@126.com (X.W.)

[3] Shandong Provincial Key Laboratory of Water Pollution Control and Resource Reuse, School of Environmental Science and Engineering, Shandong University, Qingdao 266237, China; sqni@sdu.edu.cn

* Correspondence: xmzhuang@iccas.ac.cn (X.Z.); syyu@yic.ac.cn (S.Y.)

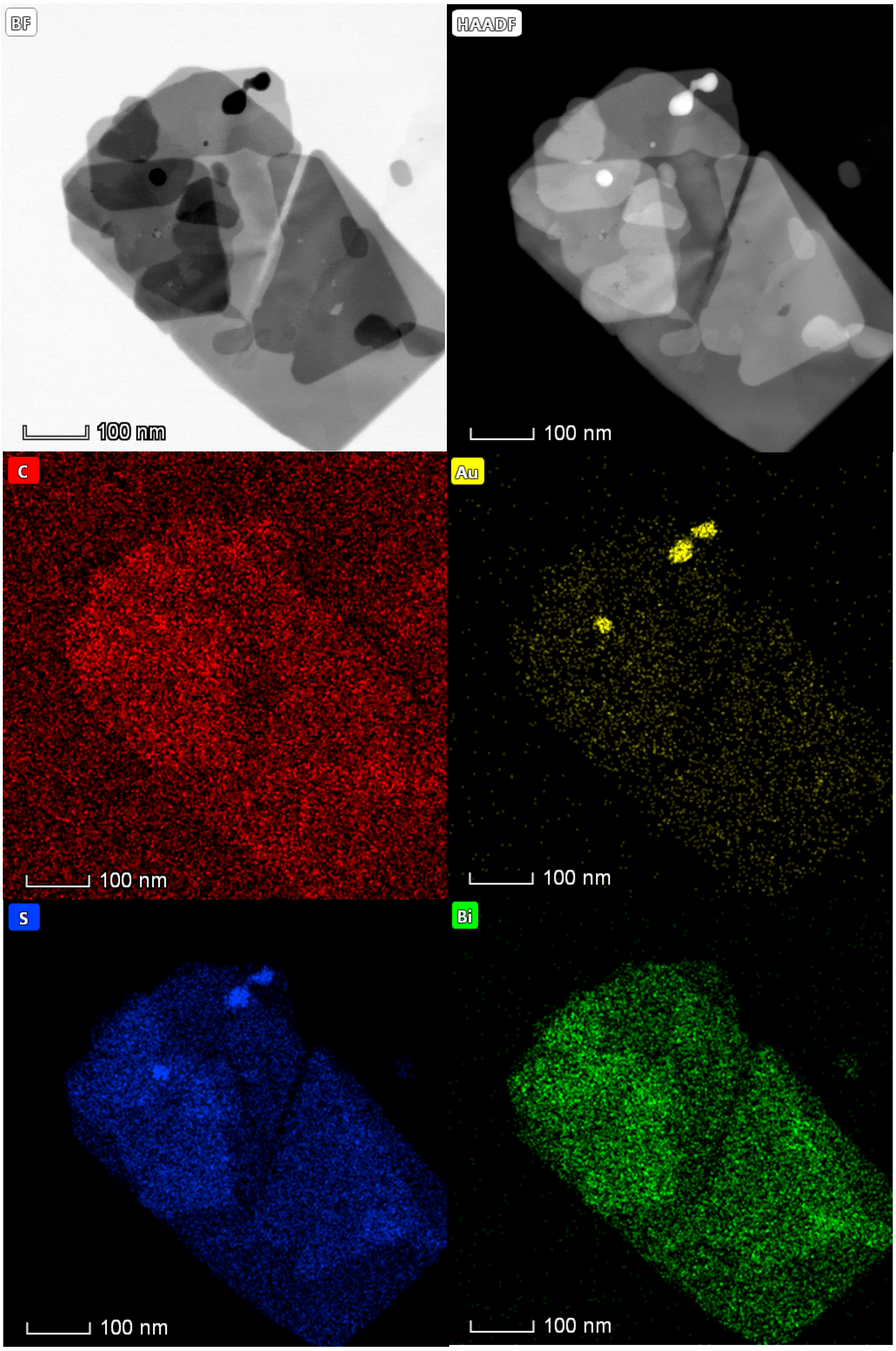

Figure S1. Elemental mapping of C, Au, S, and Bi element distribution, respectively.

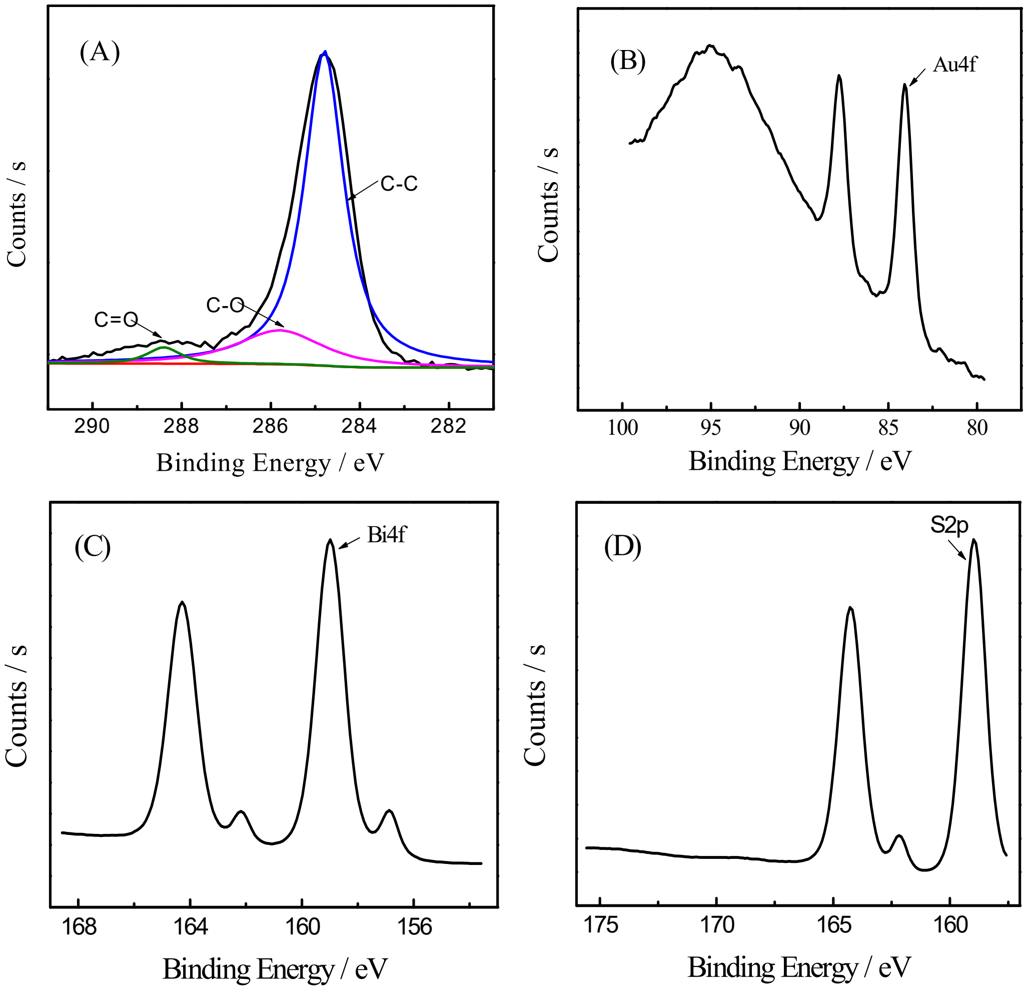

Figure S2. XPS survey spectrum of (**A**) C1s (**B**) Au4f (**C**) Bi4f and (**D**) S2p.

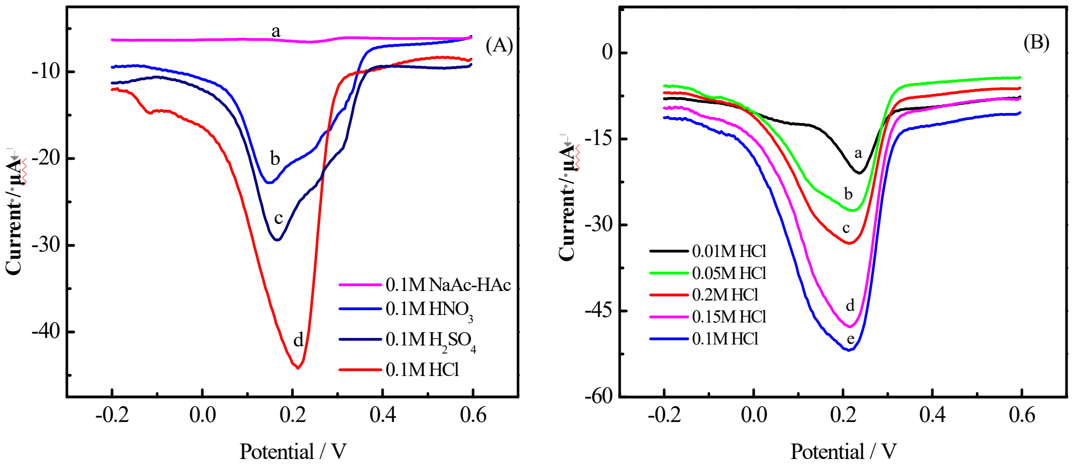

Figure S3. (**A**) SWV measurements of 5 μM Fe(III) at Au-BiNPs/SH-GO/GCE in different electrolytes. 0.1 M HAc-NaAc (a), 0.1 M HNO_3 (b), 0.1 M H_2SO_4 (c) and 0.1 M HCl (d)； (**B**) Effect of the concentration of HCl solution on the peak of Fe(III) at Au-BiNPs/SH-GO/GCE.
